# Supplementary material for: Mutation of Signal Transducer and Activator of Transcription 5 (STAT5) Binding Sites Decreases Milk Allergen αS1-Casein Content in Goat Mammary Epithelial Cells
Source: Foods. 2022 Jan 26;11(3):346. doi: 10.3390/foods11030346 (PMC8834060; doi:10.3390/foods11030346)
Supplement: Supplementary file 1 [file foods-11-00346-s001.zip › foods-1564273-supplementary/Original Images for Electropherograms.pdf]

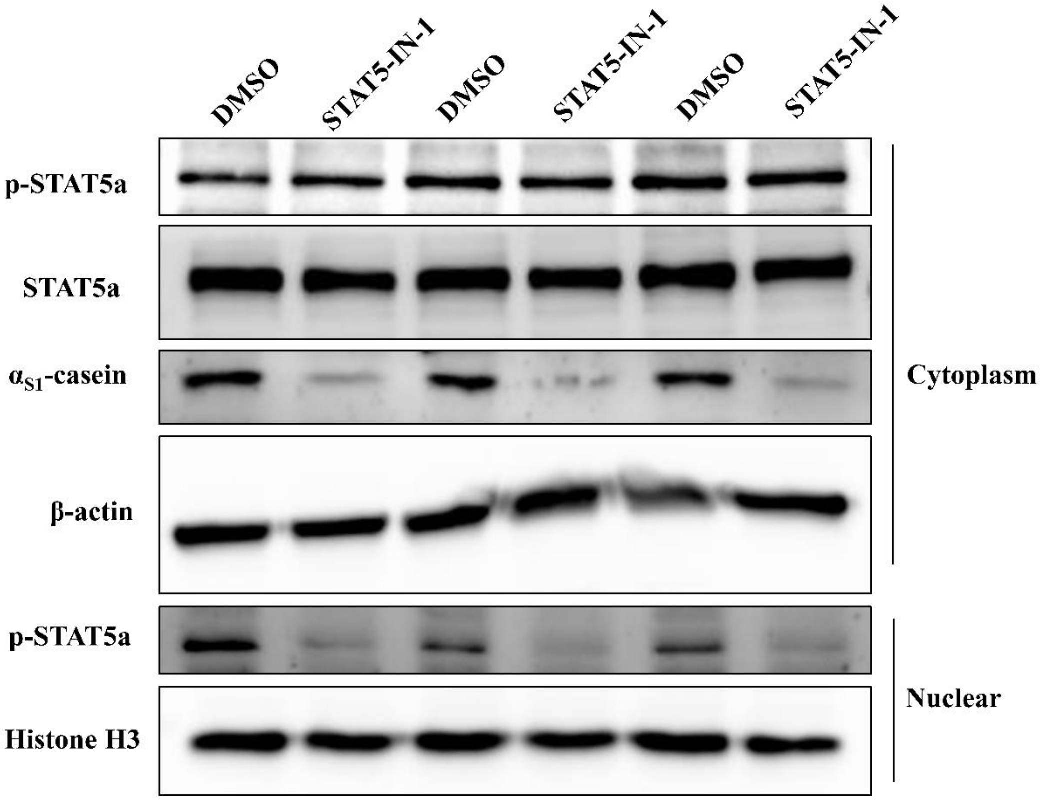

The image of the full western blot of Figure 3E. Effects of STAT5 on the expression of α_{S1}-casein. GMEC were treated with STAT5-IN-1 (200 μM) or DMSO. At 48 h treatment, protein abundance of p-STAT5a and α_{S1}-casein was measured.

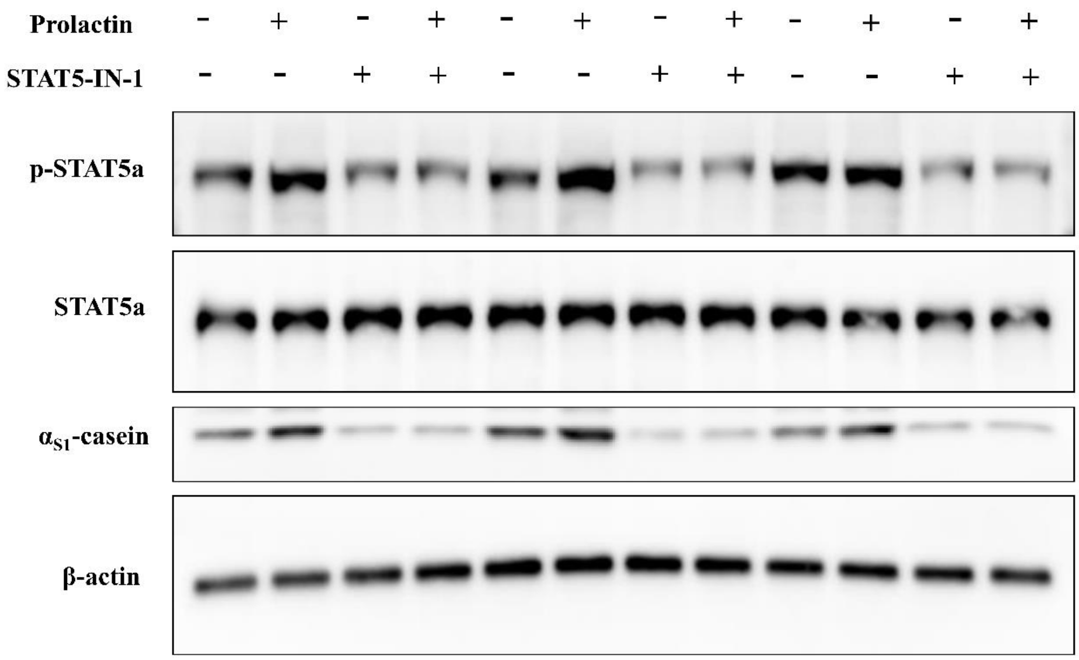

The image of the full western blot of Figure 6C. Effects of prolactin on the expression of α_{S1}-casein. GMEC seeded in culture medium were incubated with STAT5-IN-1 (200 μM, or DMSO) followed by prolactin (2 mg/L) treatment for 48 h, then total protein was extracted.

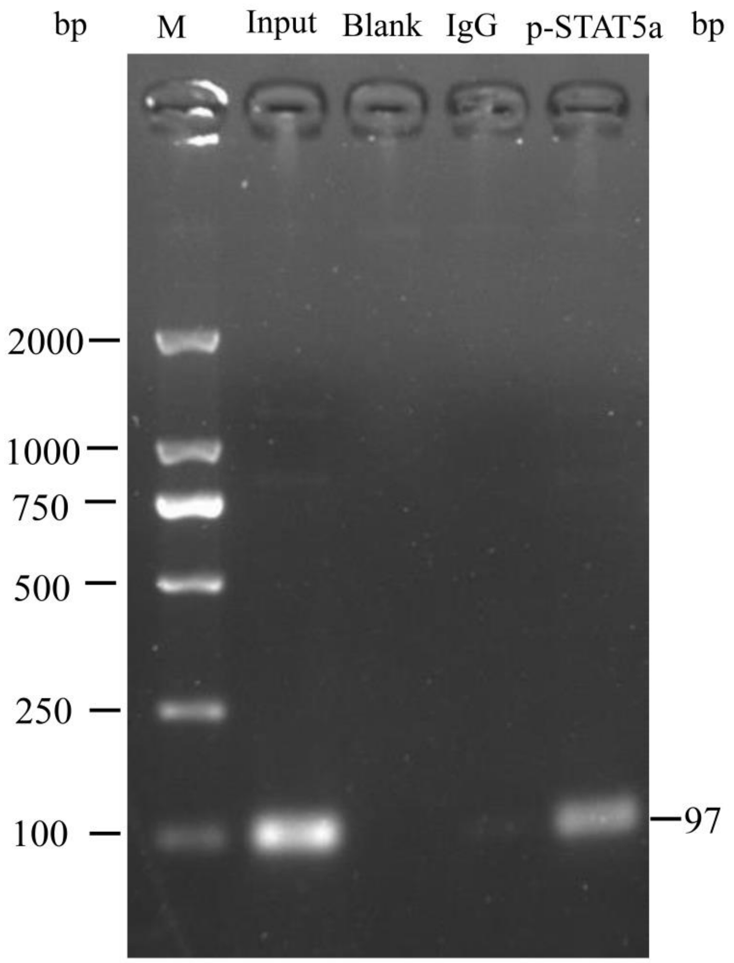

The image of the full agarose electrophoresis of Figure 7A. Transcription of *CSN1S1* gene is regulated by direct STAT5 binding to GAS sites in GMEC. GAS1 binding sites in *CSN1S1* promoter region were recognized by STAT5 directly using ChIP-PCR analysis.

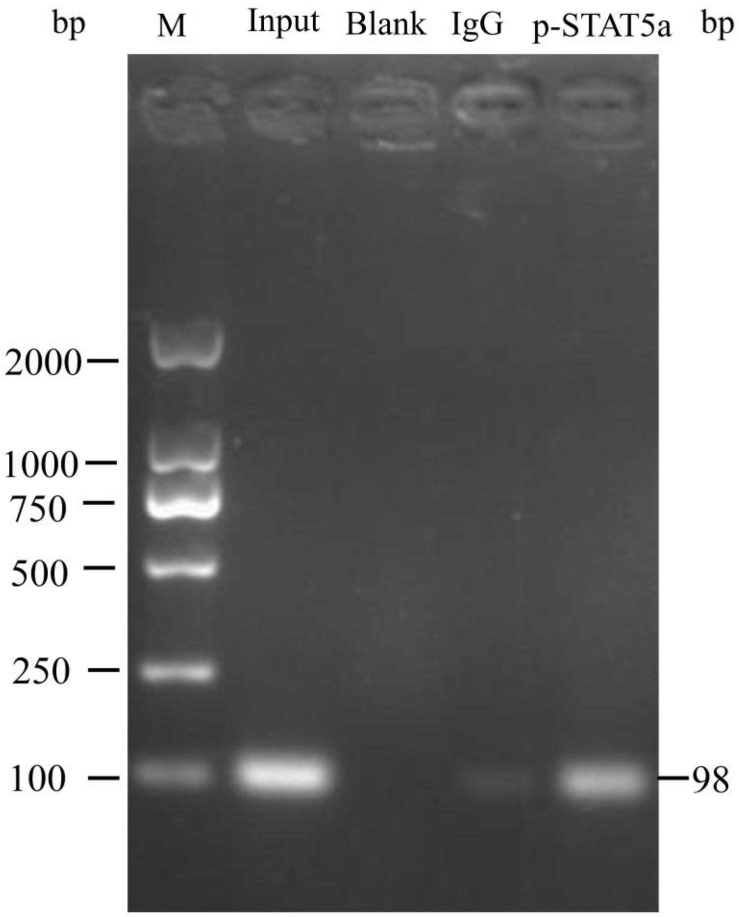

The image of the full agarose electrophoresis of Figure 7C. Transcription of *CSN1S1* gene is regulated by direct STAT5 binding to GAS sites in GMEC. GAS2 binding sites in *CSN1S1* promoter region were recognized by STAT5 directly using ChIP-PCR analysis.

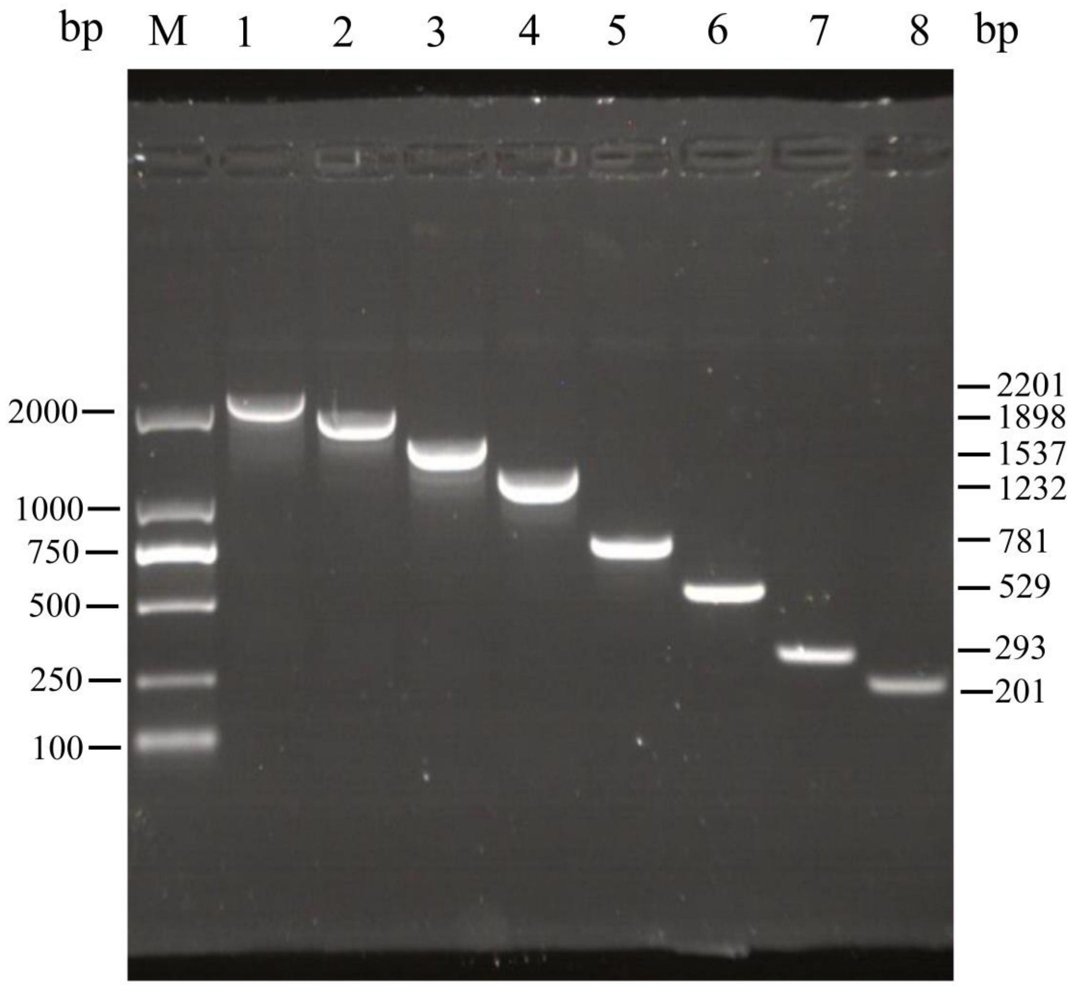

The image of the full agarose electrophoresis of Supplemental Figure S1A. Construction of deletion fragments pGL3-Basic vectors of goat *CSN1S1* gene promoter. Deletion fragment (-2018/+183, -1715/+183, -1354/+183, -1049/+183, -598/+183, -346/+183, -110/+183, -18/+183 bp) cloning of *CSN1S1* promoter.

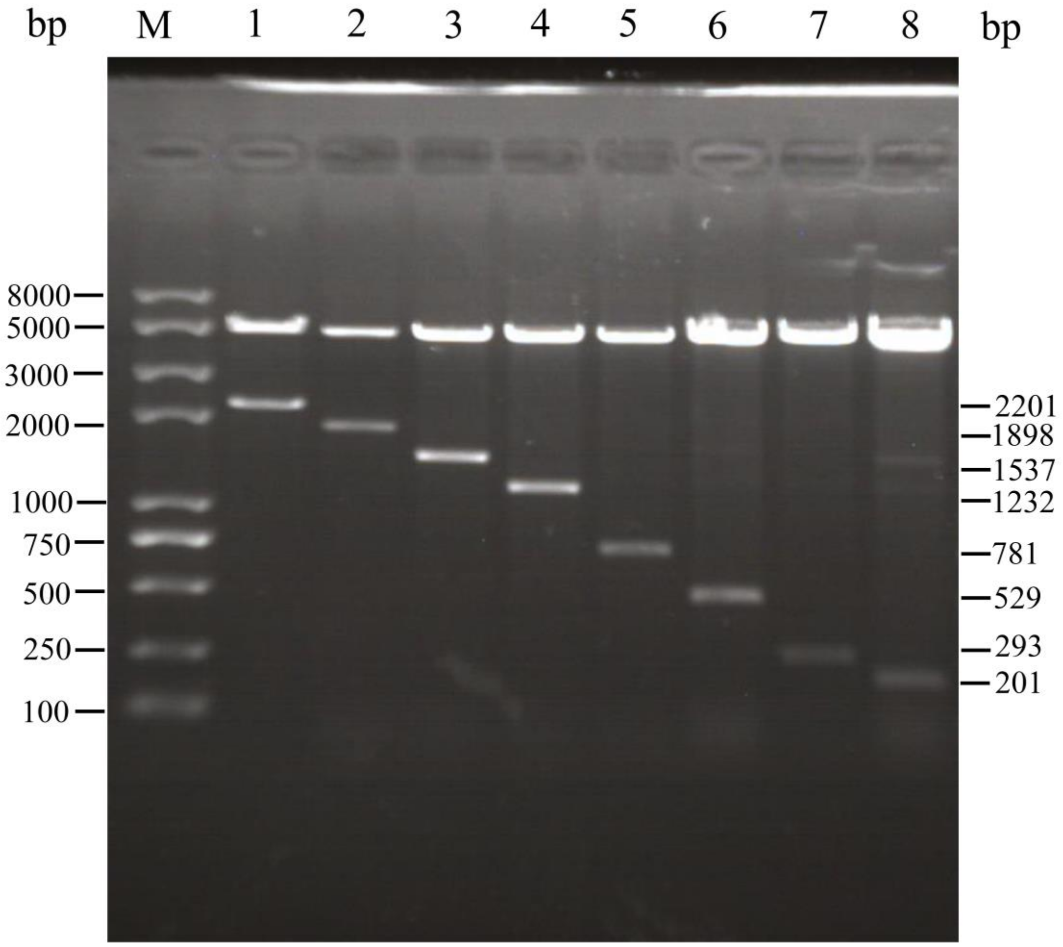

The image of the full agarose electrophoresis of Supplemental Figure S1B. Construction of deletion fragments pGL3-Basic vectors of goat *CSN1S1* gene promoter. Double enzyme digestion of recombinant plasmids of *CSN1S1* promoter fragments.
