# Supplementary material for: Mutation of Signal Transducer and Activator of Transcription 5 (STAT5) Binding Sites Decreases Milk Allergen αS1-Casein Content in Goat Mammary Epithelial Cells
Source: Foods. 2022 Jan 26;11(3):346. doi: 10.3390/foods11030346 (PMC8834060; doi:10.3390/foods11030346)
Supplement: Supplementary file 1 [file foods-11-00346-s001.zip › foods-1564273-supplementary/Supplementary Files/Supplemental Material.pdf]

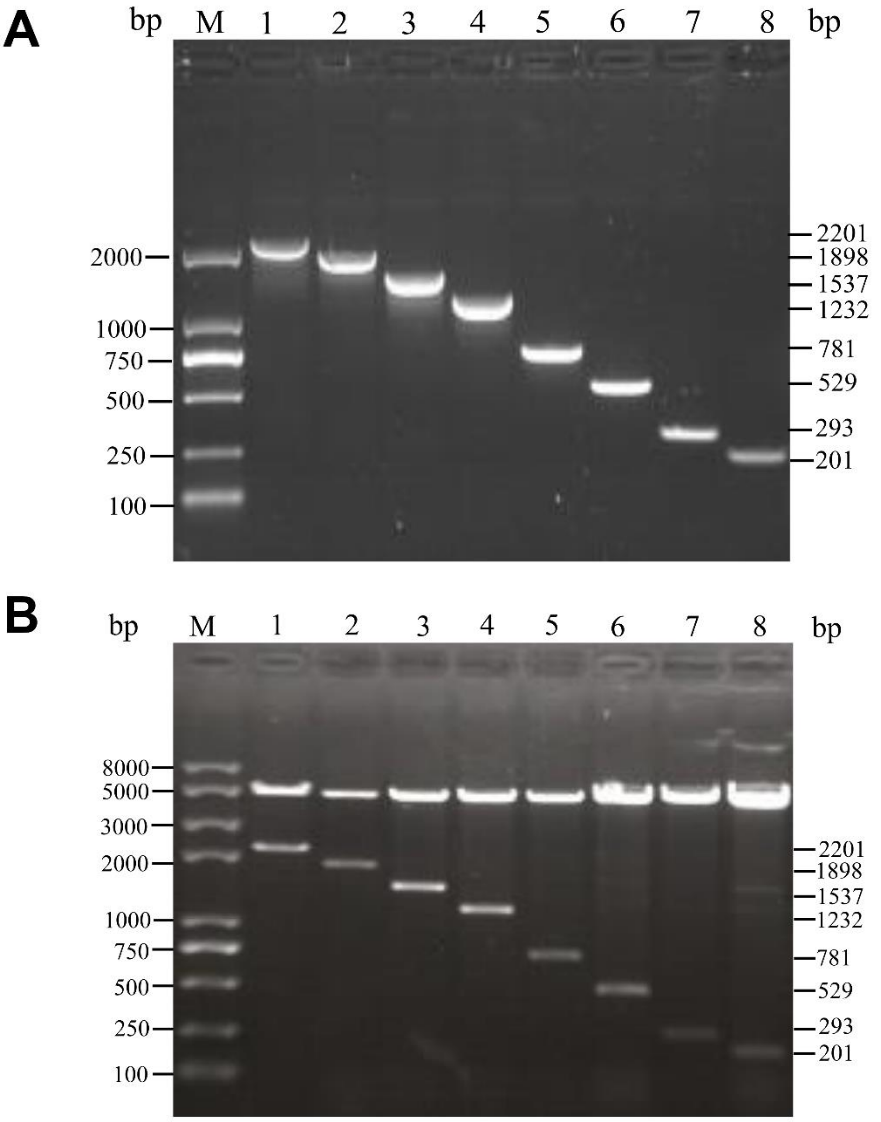

Supplemental Figure S1. Construction of deletion fragments pGL3-Basic vectors of goat *CSN1S1* gene promoter. (A) Deletion fragment (-2018/+183, -1715/+183, -1354/+183, -1049/+183, -598/+183, -346/+183, -110/+183, -18/+183 bp) cloning of *CSN1S1* promoter. (B) Double enzyme digestion of recombinant plasmids of *CSN1S1* promoter fragments.

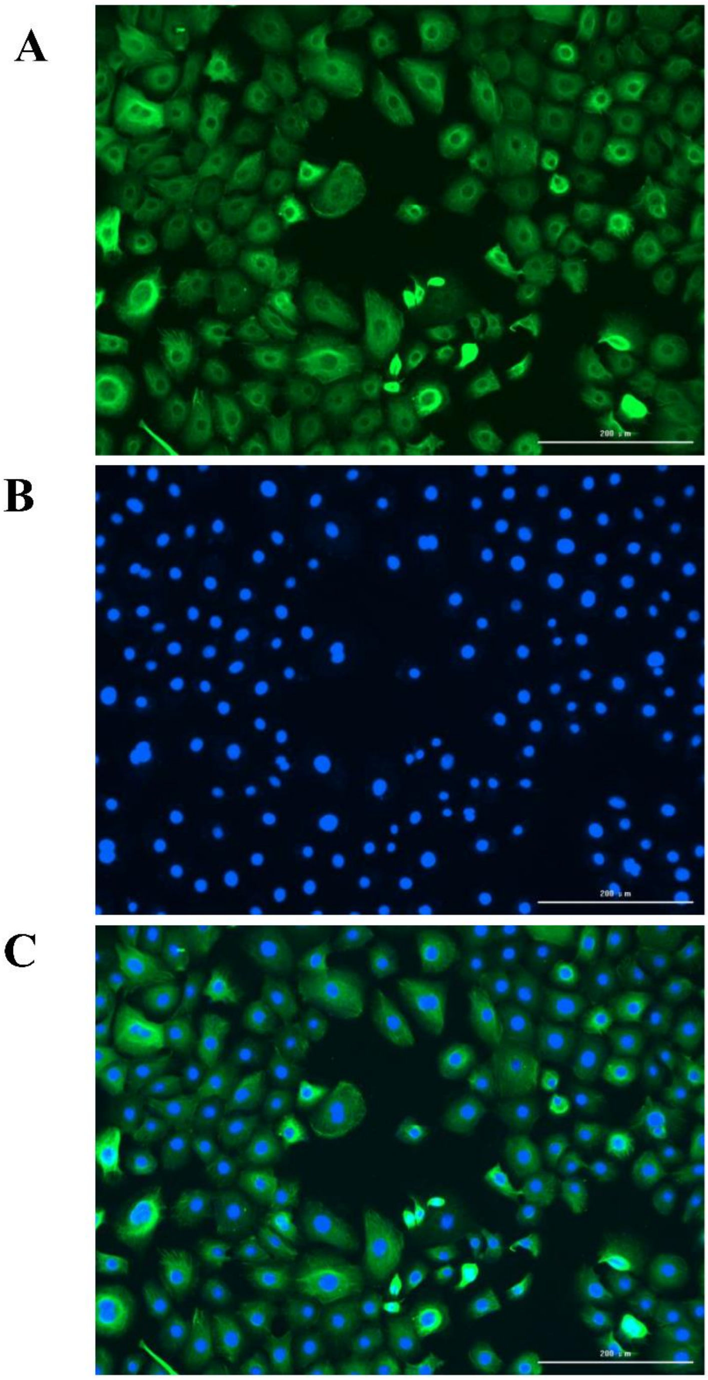

Supplemental Figure S2. Identification of GMEC by immunofluorescence with Cytokeratin 18. (A) Immunofluorescence with Cytokeratin 18. (B) DAPI staining. (C) Merge of (A) and (B). Scale bar is 200 μm.
